# A Manual of Clinical Medicine, and Physiological Diagnosis

**Published:** 1855-10

**Authors:** 


ART. V.—*A Manual of Clinical Medicine, and Phgsiological Diagnosis.* By J. H. TANNER, M. D., Licentiate of the Royal College of Physicians; Physician to the Hospital for Women, etc., etc. To which is added *The Code of Ethics of the American Medical Association.* Philadelphia: Blanchard & Lea. 1855. 12 mo., pp. 252.

CHOMEL'S Elements of General Pathology, translated by Drs. OLIVER and MORLAND, and published by Ticknor & Co., in 1848, although calculated to be one of the most useful works ever issued found its way into the hands of but few practitioners or students south of Mason and Dixon's line. How many north of this boundary profited by its publication we have no means of knowing. We believe, however, that a second edition has never been called for in this country notwithstanding the original has reached its fourth edition in Paris.

On some account we regard Chomel's, or a similar work as indispensable to the student, and of no mean importance to the physician. We believe if it had been more generally read that the heavy harangues or mumbling prelections ridiculously called clinical lectures would often have assumed another and a better form, and clinical medicine have in every instance been rendered far more attractive both to those who labor to teach and those, less fortunate, who find it a labor to learn.

The work whose title forms the caption of this article is constructed measurably after the model of that of Chomel. It is, however, a manual and we are from principle averse to all manuals, compends, hand-books and remembrancers whatever. Yet, in view of the very limited circulation already referred to of Chomel's Elements, and the great hiatus that confessedly exists in this department of medical literature, we think Tanner's Clinical Medicine a well timed publication, and cheerfully recommend it to our readers.

